# A fabric-based wearable sensor for continuous monitoring of decubitus ulcer of subjects lying on a bed

**DOI:** 10.1038/s41598-023-33081-7

**Published:** 2023-04-08

**Authors:** Soyeon Lee, Seung-Rok Kim, Kun-Hoo Jeon, Jun-Woo Jeon, Ey-In Lee, Jiwan Jeon, Je-Heon Oh, Ju-Hyun Yoo, Hye-Jun Kil, Jin-Woo Park

**Affiliations:** 1 Department of Materials Science and Engineering, Yonsei University, 50 Yonsei-Ro, Seodaemun-Gu, Seoul, 03722 Korea; 2 Asen Company, Seoul, 03722 Republic of Korea

**Keywords:** Biotechnology, Materials science

## Abstract

For multifunctional wearable sensing systems, problems related to wireless and continuous communication and soft, noninvasive, and disposable functionality issues should be solved for precise physiological signal detection. To measure the critical transitions of pressure, temperature, and skin impedance when continuous pressure is applied on skin and tissue, we developed a sensor for decubitus ulcers using conventional analog circuitry for wireless and continuous communication in a disposable, breathable fabric-based multifunctional sensing system capable of conformal contact. By integrating the designed wireless communication module into a multifunctional sensor, we obtained sensing data that were sent sequentially and continuously to a customized mobile phone app. With a small-sized and lightweight module, our sensing system operated over 24 h with a coin-cell battery consuming minimum energy for intermittent sensing and transmission. We conducted a pilot test on healthy subjects to evaluate the adequate wireless operation of the multifunctional sensing system when applied to the body. By solving the aforementioned practical problems, including those related to wireless and continuous communication and soft, noninvasive, and disposable functionality issues, our fabric-based multifunctional decubitus ulcer sensor successfully measured applied pressure, skin temperature, and electrical skin impedance.

## Introduction

**Figure 1:**
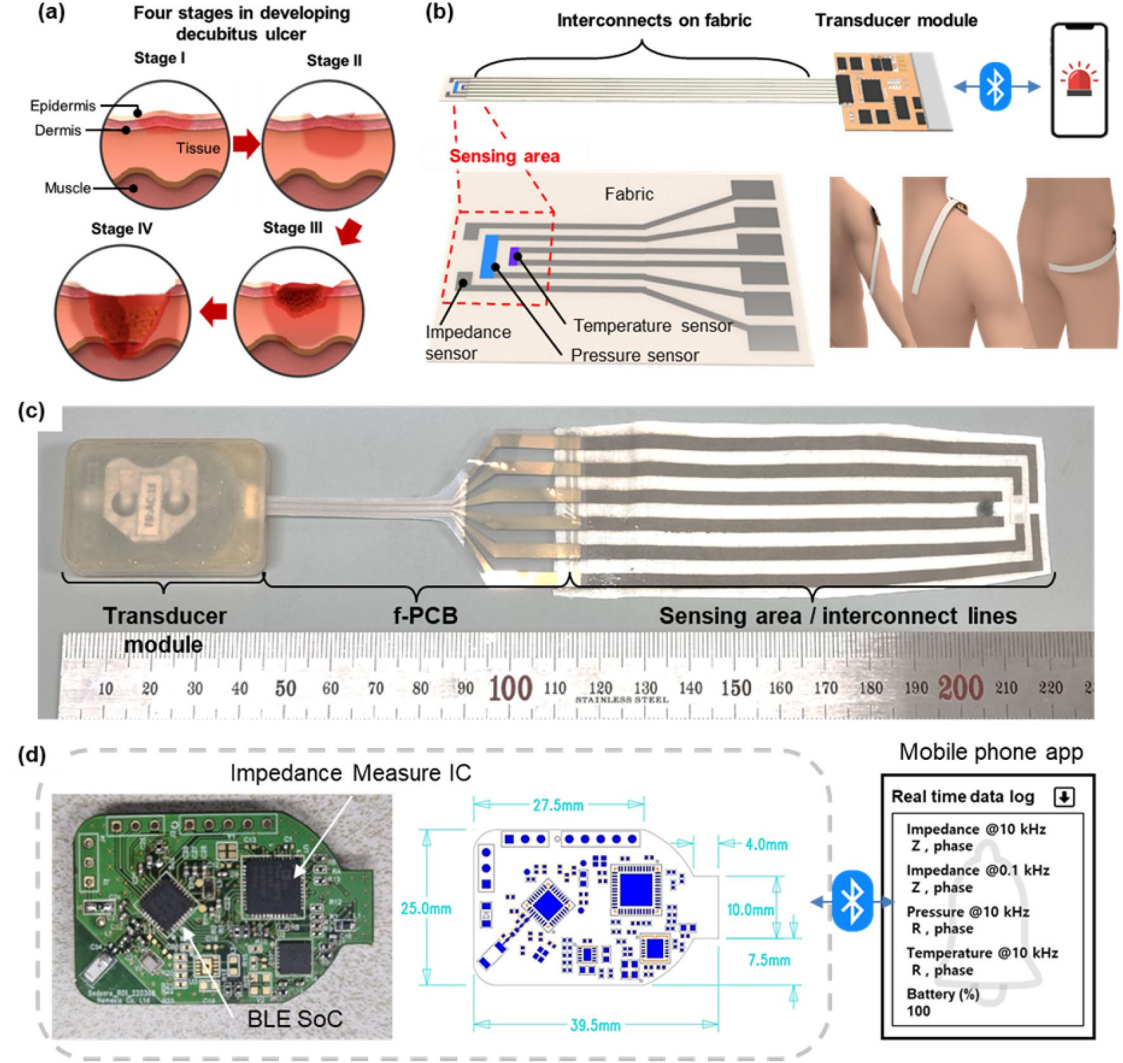
(**a**) Four developmental stages of decubitus ulcers. (**b**) Schematic illustration of wirelessly communicating fabric-based bedsore sensors. (**c**) Optical image of wirelessly communicating fabric-based bedsore sensors, including the sensing area, interconnect lines, and communication module. (**d**) Optical image of the printed circuit board (PCB) module, including the *Z*-measuring integrated circuit (IC) and Bluetooth low energy (BLE) system on a chip (SoC), and a snapshot of the mobile phone app.

With the increase in the aging population, the increasing number of long-term bedridden patients and their high morbidity rate have attracted the focus of socioeconomic and public healthcare organizations^1^. Among the challenging issues related to these patients, such as decubitus ulcers, diabetes mellitus, and medication addiction, chronic wounds caused by prolonged, localized pressure (*P*) on the body are one of the most cumbersome problems because of the resulting contamination and infection^2,3^. The developmental process of the decubitus ulcer can be divided into four stages (Fig. 1a): the decubitus ulcer in Stage I or II is reversible and easily healable^4^. However, once the ulcer reaches Stage II, it easily and rapidly develops into Stages III and IV, at which point the ulcer is very difficult to cure with the exposure of the inner skin to ambient air^4^. Therefore, detecting this early-stage transition is critical for saving patients from multiple surgical operations^2^.

Exploiting skin-like wearable sensor technology is the most effective approach for monitoring the early-stage transition of decubitus ulcers^5,6^. For accurate measurement, these sensors should simultaneously detect multiple disease-related indicators^2^. Many disease-related indicators have been studied, such as the duration of the applied pressure (*P*)^7,8^, local temperature (*T*)^9,10^, relative humidity^11,12^, blood flow^13,14^, or skin impedance (*Z*) variation^15,16^, to quantify the assessment of ulcers. Damage or death of a cell under the pressured skin reduced impedance value caused by the cell losing its membrane structure and becoming rich with conducting path ^15^. However, there are inconsistent patterns in skin temperature or blood flow variation around the pressured skin, showing unclear relationship with the biomarker^17–20^.

Although various sensing targets for skin decubitus ulcers have been proposed, many practical issues have not yet been solved, such as the issues of wireless powering and data transmission for continuous sensing^7,21,22^, softness for minimal mechanical mismatch with the skin^1,23^, noninvasiveness for long-term usage^24,25^, and disposability to obtain a low-cost and hygienic sensor^3,26^.

Specifically, in consideration of the aforementioned softness and hygienic issues, commercially available fabrics or bandages can be an attractive solution for constructing a real-time skin monitoring system that patients can wear in everyday life^3,26^. Additionally, exploiting mature remote communication technologies, such as Bluetooth or ZigBee, can allow sensing systems to continuously monitor any tiny abnormal signal variations of skin indicators^7,21,22^. Moreover, the collected data recorded by the sensors can be intermittently transmitted to remote medical staff as spatially distributed information because every spot on the body with prolonged *P* can be at risk of developing a decubitus ulcer.

In our previous works^2^, an e-fabric multifunctional sensor that simultaneously monitored *P*, *T*, and *Z* in a nude mouse model successfully detected the critical period before the development of early-stage decubitus ulcers, which was verified by various biophysical evidence from mouse skin ischemia. Ultimately, to confirm the applicability of the e-fabric multifunctional sensor for the early detection of decubitus ulcers in potential patients, we need to establish and evaluate a platform that allows the accurate monitoring of human skin conditions as well as the transfer of real-time data to medical staff.

In this work, we developed a miniaturized impedance sensing module for the collection and transmission of data from sensors to a mobile phone app. The completely integrated wearable decubitus ulcer sensors on simple cotton fabric could wirelessly and continuously monitor the external applied *P*, *T*, and electrical *Z* of the skin to detect potential decubitus ulcers. Moreover, disposable sensors on fabric and wireless electronics based on conventional analog circuitry were smartly integrated into a plastic slot with printed interconnects on it, making it possible for the electronic module to be reused with the application of new fabric strips with multiple sensors. Patients and caregivers can be notified through the wireless module when the skin conditions become abnormal. Here, we pilot-tested our e-fabric-based bedsore sensor on healthy subjects lying on a bed to provide timely information to provide proof of concept. Our fabric-based sensors can be applied on the bodies of patients at risk for developing bedsores in daily life, allowing patients and caregivers to be warned of early-stage decubitus ulcers.

## Results and discussion

### Concept of the wireless multifunctional e-textile skin ulcer sensor

**Figure 2:**
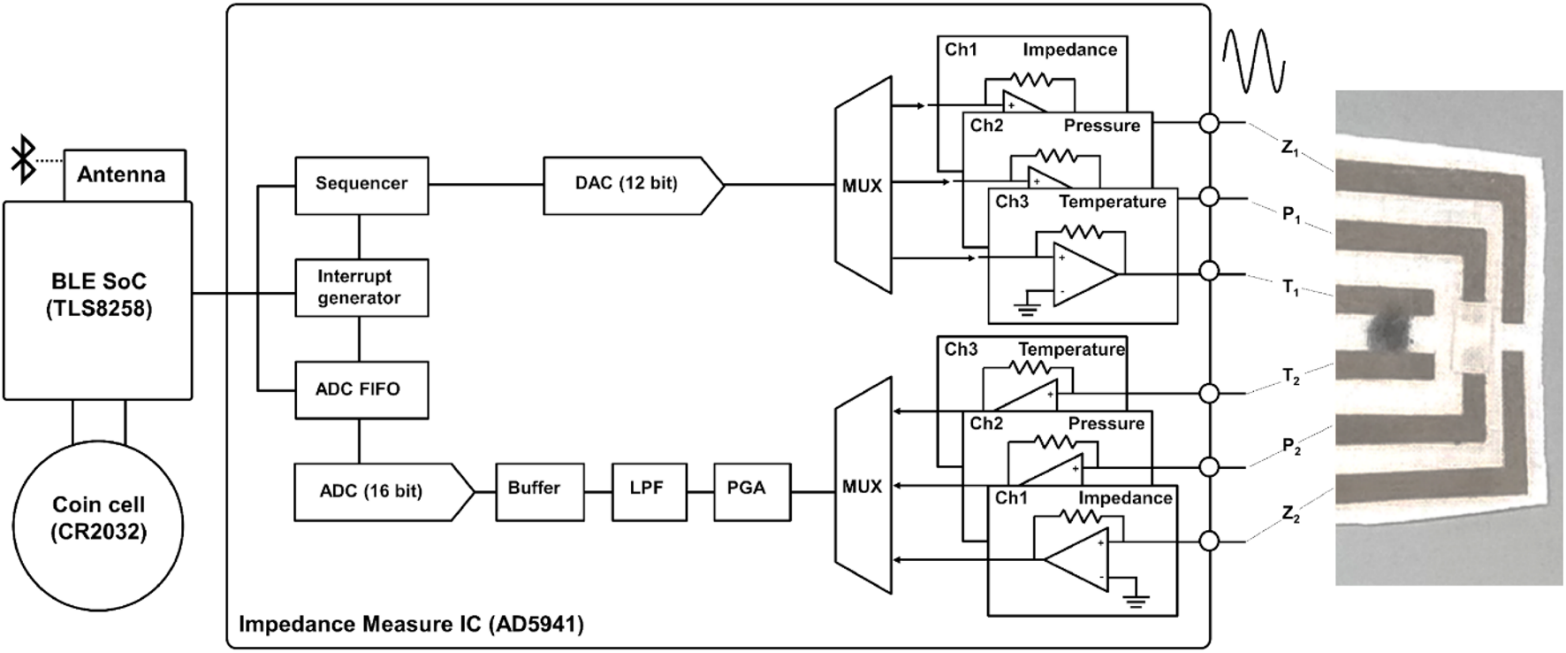
System components and configuration of the wirelessly communicating multifunctional e-textile skin ulcer sensor.

**Figure 3:**
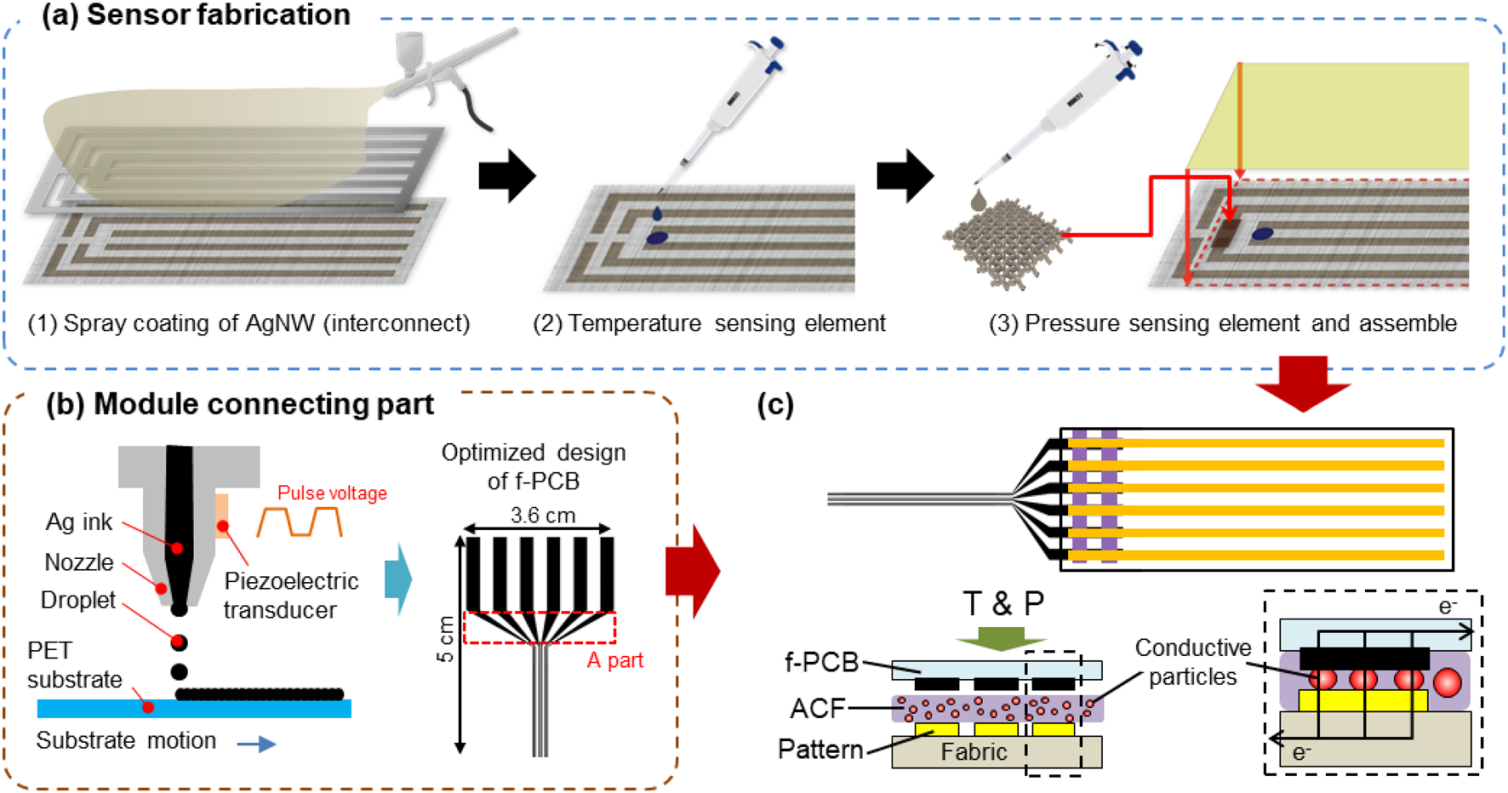
Schematic images of the (**a**) sensor fabrication, (**b**) module connecting part fabrication, and (**c**) total integration.

Our wireless decubitus ulcer sensor can be divided into three functional parts: (1) the sensing area and interconnects on the fabric, (2) the module connecting part on the polyethylene terephthalate (PET) substrate, and (3) the wireless measurement module (Fig. 1b,c). Most of the sensors and interconnects were fabricated on the fabric to achieve permeability to gases and moisture possibly released from the potential ulcer areas, which prevented further wound progress due to restricted air circulation near the contact area. The *P-* and *T*-sensing elements were assembled on top of silver nanowire (AgNW) electrode interconnects on the fabric (AgNW/fabric). The exposed paired electrodes at the very top of the sensing area functioned as the *Z*-sensing element. The detailed components of the sensing elements and the fabrication method are described the Methods section and are shown in Figs. 2 and 3.

As shown in Fig. 1b, the sensing area can be placed on any part of the human body, such as the elbow, heel, ear, and scapula, due to its conformable and tunable features. The length of the interconnect on the fabric, which can be applied to any part of the body regardless of its size, can be reliably modulated up to 15 cm on a laboratory scale, considering that the full half-length of the periphery of the human elbow is within 15 cm (Fig. 1c). To successfully achieve an electrical connection between the wireless measurement module and the sensing elements on the fabric, we designed a flat flexible cable (FFC) through the inkjet printing of Ag ink on PET substrate. Compared to commercially available parallel FFCs, our FFC has both wide and narrow lines to connect the sensors and the 8-pin connector of the measurement module.

As shown in Fig. 1d, the measurement module was composed of a *Z*-measuring integrated circuit (IC) and Bluetooth low energy (BLE) system on a chip (SoC) on a printed circuit board (PCB), with dimensions of 39.4 mm × 25.0 mm × 6.44 mm and a weight of 3.85 g without a battery. The overall module can be continuously operated for over 24 h using a 3 V coin-cell battery. The developed mobile phone app can display the data (*P*, *T*, and *Z*) every 10 s. With our wireless multisensor system, the patient and caregiver can be notified before the development of stage II decubitus ulcers, which was clearly proven in our previous animal model studies^2^. The doctor can monitor and advise the patient about the everyday skin conditions at potential ulcer sites.

### System configuration

As illustrated in Fig. 2, our sensing system employed a BLE SoC (TLS8258) as the main application chip powered by a coin-cell (CR2032). The BLE SoC controlled the *Z*-measuring IC (AD5941) and acquired the sensors' data. Alternating current signals of 1 kHz, 2.17 V_pp_ were generated through a digital-to-analog converter (DAC) and distributed sequentially to the sensors through a multiplexer (MUX). The signals passing through the sensors were sent to an analog-to-digital converter (ADC) through the MUX, a programmable gain amplifier (PGA), and a low-pass filter (LPF). We obtained the *Z* values of the sensors by using the amplitude ratio and the phase shift of the sinusoidal wave generated before and after the sensor. Every 10 s, the measured *Z* data were wirelessly transmitted through the antenna using the BLE protocol to a nearby mobile phone with a custom data acquisition app. We used a multiplexor to operate the sensors sequentially to avoid signal coupling or distortion caused by intrinsic AC inputs, signal collision, and parasitic effects. Furthermore, in accounting for the matters of parasitic capacitance or inductance, we evaluated the sensors’ performance using our developed wireless modules instead of the commercially available measurements.

### Fabrication and evaluation of the multifunctional sensor

Figure 3 shows the fabrication processes of our sensor. As briefly described and shown in Fig. 1, the sensor was fabricated in two independent steps (the fabrication of the sensor and module connecting part, respectively). For the fabrication of the sensing element on the fabric, AgNWs were spray-coated on cotton (50 twill weave), as plain-woven cotton was selected as the best candidate for fabricating AgNW electrodes, as verified in previous reports^2^. As shown in Fig. 3a, a stainless steel (SUS) mask was used to pattern the interconnect lines, which provided a 3 mm electrode width and space. The 3 mm electrode width was designed to achieve a uniform and tunable electrical performance along the line with a length of 10 ~ 15 cm, and the 3 mm space was designed to prevent an electrical short between the lines.

On the AgNW/fabric interconnect, a poly(3,4-ethylenedioxythiophene):polystyrene sulfonate (PEDOT:PSS)/multiwalled carbon nanotube (MWCNT) composite in DI water was drop-cast on the center of the interconnect lines, which functioned as the *T*-sensing element. The composite had a negative *T* coefficient, indicating that the electrical tunneling increased as *T* increased, decreasing the electrical resistance (*R*) of the *T*-sensing element. The mixing of MWCNTs can stabilize the *T*-responsive *R* change of PEDOT:PSS, as described in our previous report^2^.

After the formation of the *T*-sensing element, the *P*-sensing element was assembled on top of the two interconnect lines using Tegaderm film. The *P*-sensing element was independently fabricated by drop-casting an extremely dilute AgNW solution (0.0792 wt%) on the as-received cotton fabric, with a high *R* of 1000 Ω per 1 cm. A contact *R* could be determined by the intersecting conductive area between the *P*-sensing element and the interconnect lines, allowing the *P*-induced contact *R* variation to be monitored through the external circuit.

As shown in Fig. 3b, our customized FFC was fabricated using an inkjet printing machine (EPSON-1170), of which the nozzle tip dropped the commercially available Ag ink on the surface-treated PET substrate (Novele™ pack). Here, the minimum resolution of our inkjet printing was 0.2 mm in the printing direction and 0.5 mm in the perpendicular direction. Therefore, we designed a spacer for diagonal geometry (A part) within 1 cm for a reliable electrical connection. Finally, as shown in Fig. 3c, the bottom-most part of our sensors on the fabric and the topmost part of the FFC were connected through an anisotropic conductive film (ACF). The ACF helped form the vertical conducting path between the fabric and PET with a *P* of 40 kPa and *T* of 160 °C, which was optimal for our polymer-based substrates.

**Figure 4:**
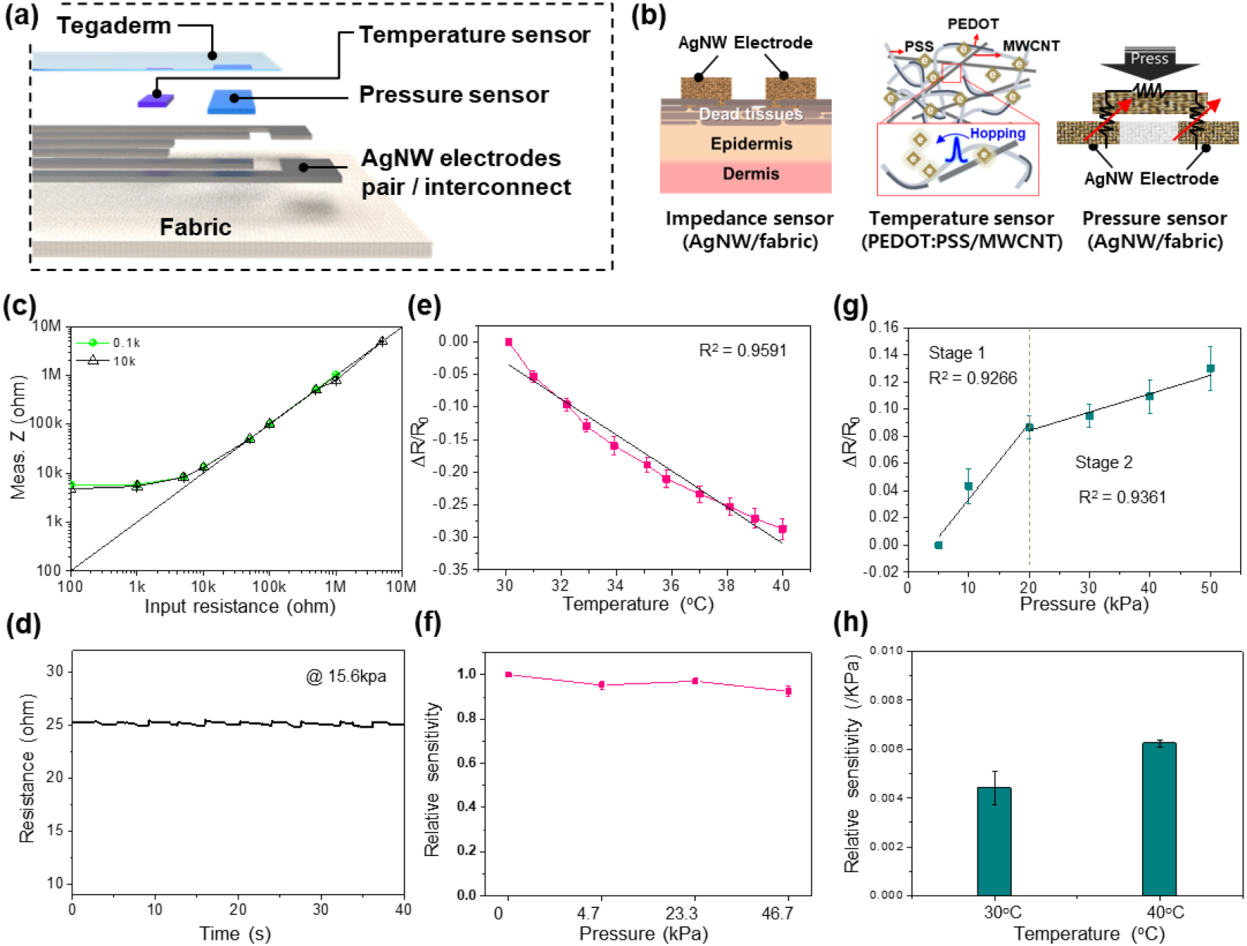
Performance of the multifunctional e-textile skin ulcer sensor. Schematics of the (**a**) device architecture and (**b**) sensing mechanisms of the skin *Z*, *T*, and *P* sensors. (**c**) *Z*-sensing performance of the AgNW/fabric-based *Z* sensor. (**d**) *R* of the AgNW/fabric interconnects measured in the cyclic loading test. (**e**) *T*-sensing performance of the PEDOT:PSS/MWCNT-based *T* sensor. (**f**) Relative sensitivity changes of the *T* sensor under different *P* levels from 0 to 46.7 kPa. (**g**) *P*-sensing performance of the AgNW/fabric-based *P* sensor. (**h**) Relative sensitivity changes of the *P* sensor under different *T* values of 30 and 40 °C.

The sensing performance and reliability of our sensors on the fabric were investigated, as shown in Fig. 4. The paired electrodes at the very top of the sensing area functioned as a *Z* sensor, by which the AC electric field supplied by the two electrodes could penetrate through skin so that the condition of cells below the dermis and epidermis could be monitored (Fig. 4b). Considering that a frequency-dependent electric field deeply penetrates through skin, we designed the module to obtain the *Z* data at applied AC frequencies of 0.1 and 10 kHz to detect the death or damage of cells at the dermis layer and epidermis layer, respectively^27,28^.

To evaluate the *Z*-sensing accuracy of our *Z* sensor combined with the wireless modules, various input *R* values ranging from 100 Ω to 5 Mohm were detected by connecting *R* to our *Z* sensor. Figure 4c shows that the measured *Z* (Meas. *Z*) values from the module at frequencies of 0.1 and 10 kHz were linearly correlated with the input *R* values ranging from 5 kohm to 5 Mohm, verifying the capability of our *Z* sensor and wireless module to detect the skin *Z* value, which normally ranges from 10 kohm to 1 Mohm. Because the value of *Z* varies with the size, geometry, and arrangement of electrodes^29^, the performance of our impedance sensor can be determined by comparing our data with the impedance sweeping data obtained through electrochemical impedance spectroscopy (EIS), as shown in Fig. S1.

With the development of the geometrical design of our AgNW/fabric electrodes and the extendable circuit element in the wireless module, our *Z* sensor produced more accurate *Z* information for the detection of smaller and larger skin *Z* variations beyond the measuring *R* range from 1 kohm to 5 Mohm of the AD5941 chip integrated in our wireless module^30^. Additionally, the robustness of the *Z* sensor and interconnect lines was investigated by the cyclic loading and unloading of a 15 kPa weight on the center of our AgNW/fabric (Fig. 4d). The *R* barely changed, indicating the robustness of our AgNW electrode on the fabric.

On the other hand, the *T-* and *P*-sensing performance was investigated by evaluating the relative *R* change (∆*R*/*R*_0_) in response to *T* and *P* changes, respectively. The ∆*R*/*R*_0_ of the *T* sensor was linearly dependent on the *T* change, with a slope of -0.0276/°C and R^2^ of 0.9591 in a *T* range from 30 to 40 °C (Fig. 4e). The initial *R* (*R*_0_) was the *R* under the temperature of 30 °C, and it ranges 123.3 ± 8.60 kohm. Additionally, the *T* sensor showed stable *T* responses under *P* changes from 0 to 46.7 kPa, with a sensitivity change of less than 10% (Fig. 4f).

The operating mechanism of the *P* sensor involved the contact *R* change between the floating conducting fabric and paired electrodes, as shown in Fig. 4b. Therefore, the initial *R* (*R*_0_) was the *R* under the loading of 5 kPa weight for the reliable evaluation of the floating *P*-sensing element. The sensitivity of the pressure sensor is given by *S* = δ(∆*R*/*R*_0_)/δ*P*, where ∆*R* is the change in *R*(*R*-*R*_0_). The *R*_0_ of the pressure sensor is 955.75 ± 313.1Ω. The contact *R* rapidly decreased in the *P* range of 0 to 20 kPa (Stage 1, *S* = 0.00555/kPa, R^2^ of 0.9266), and the contact *R* variation rate decreased in the *P* range of 20 to 50 kPa (Stage 2,* S* = 0.00135/kPa, R^2^ of 0.9361) due to the saturated contact interface between the floating conductive element and interconnect lines. Figure 4d illustrates two linear pressure sensing stages, where stage 1 had a sensitivity of 2.5 kPa^−1^ at pressure levels from 0 to 40 kPa, while stage 2 had a sensitivity of 0.12 kPa^−1^ at pressures above 40 kPa. Additionally, the *P* sensor showed cross-sensitivity with *T*, as shown in Fig. 4h. The relative slope of the *P* sensor under the *P* level (23.3 kPa) at 30 °C increased when increasing the temperature to 40 °C. The increase in the *P* sensitivity at higher *T* was due to the negative *T* coefficient of the AgNWs. When *T* increased, the number of excited electrons inside the AgNWs decreased (due to scattering in the metallic material), and the *R* of the AgNW/fabric increased. Here, the *P*-induced decrease in the contact *R* between the AgNW/fabric significantly depressed the increased *R*, which increased the ∆*R* and relative slope.

The relative slope of the *P*-sensing element at 30 °C was 0.0044/kPa and that at 40 °C was 0.0062/kPa, indicating a change in the slope of 0.00018/kPa/°C. Assuming a change in body *T* within 3 °C, the slope change amount would be 0.00054/kPa, and the final slope would be 0.00494/kPa. For example, when the *P*-induced ∆*R*/*R*_0_ was 0.02, the slope before (0.0044/kPa) and after (0.00494/kPa) the *T* change was used to calculate *P* levels of 4.545 and 4.048 kPa, respectively. Considering that the function of the *P* sensor was to indicate abrupt changes in applied *P* over 2.5–3 kPa due to postural changes between different body positions, the cross-sensitivity of our sensor is nearly negligible. Despite the mildness of the cross-sensitivity of our system, this problem should be addressed in future work with the further development of calibration algorithms and robust material engineering. Our fabric-based sensor had proper durability proved in our previous study^2^, where the sensing performance are maintained after one day in ambient conditions and after one day when applying a 100 kPa load and 35 °C simultaneously.

Overall, in terms of reusing the circuit board with different sensors (made in a same way), the inter-sample variation can be accepted as long as the sensors are within the certain range of impedance value that is matched with the range in PCB. Still, specific calibrating coefficients will be applied differently in every sensor, and the users will be given each value. On the other hand, it is challenging to precisely position the *T*-sensing and *P*-sensing active parts at the exact distance between AgNW interconnection lines using manual drop-casting and packing methods for manufacturing. These approaches cause significant deviation in the initial resistances of the sensors; however, manufacturing automation using dispensing robots can solve this issue in our future development.

### Pilot tests of the multifunctional sensor

**Figure 5:**
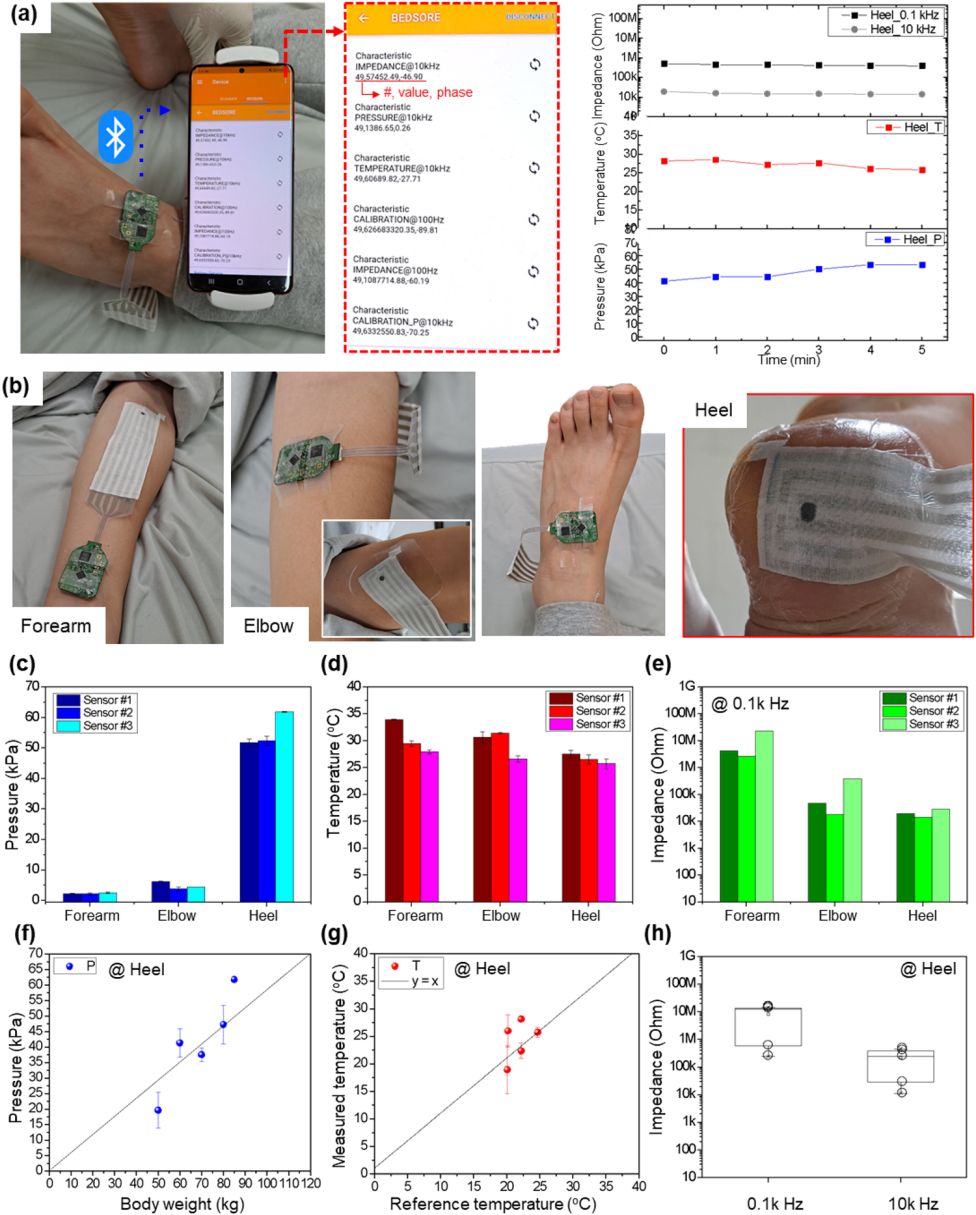
On-body test of the multifunctional e-textile skin ulcer sensor. (**a**) Optical images of the multifunctional e-textile skin ulcer sensor after mounting on the body, a snapshot of a real-time communicating smartphone application, and the signals from the sensor over 5 min. (**b**) The sensors were continuously tested on multiple sites, including the forearm, elbow, and heel. Using three samples, the (**c**) *P*, (**d**) *T*, and (**e**) *Z* of the skin were measured from multiple sites. On the heel site, *P*, *T*, and *Z* were tested with five subjects. (**f**) The relationship between the *P* values and the subjects’ body weight was assessed. (**g**) The *T* values were compared to the *T* values measured by a thermocouple. (**h**) The *Z* values were measured using frequencies of 0.1 kHz and 10 kHz.

As shown in Fig. 5, to evaluate the efficacy of our sensors in the wireless monitoring of *P*, *T*, and *Z* biosignals of healthy subjects in a lying down posture, the sensors were investigated with two different approaches: (1) the first approach involved a reliable sensor operation test wherein sensors were attached on different body areas of a subject, and (2) the second approach involved a correlation analysis between the different body weights of the subjects and their *P*, *T*, and *Z* biosignals. Five subjects with different body weights of 50, 60, 70, 80, and 85 kg were recruited for the correlation analysis. As shown in Table 1, the subject’s skin temperature and dryness at the heel position were monitored by a thermocouple temperature sensor and through tactile determination. Considering the anatomical distribution of decubitus ulcers, the high-risk areas are the heel and sacrum, and we selected the heel as our measurement area due to the ease of sensor attachment. Since this pilot test was not designed to induce decubitus ulcers but to evaluate our sensors' wireless operation on healthy subjects, we monitored three biosignals over 5 min, as shown in the graph on the right-side of Fig. 5a. The transferred data were acquired every 10 s, and here, we displayed the data obtained every 1 min to identify the trends of the variable biosignals.

**Table 1:** Information on the subjects who participated in the pilot test.

Subject	Weight (kg)	Sex	Temperature (°C)	Dryness
1	50	F	20	Dry
2	60	M	20	Dry
3	70	M	22	Dry
4	80	M	23	Wet
5	85	M	25	Wet

For the first approach, the data obtained from the heel were compared with the data obtained from the forearm and elbow, where no *P* and low *P* values were expected for these respective areas, as shown in Fig. 5b–e. The desired attachment area was wiped with an ethanol swap, and the sensor was fixed using Tegaderm adhesive. The module part was positioned near the monitoring area using SRTO bioadhesive tape. After connecting the module to the sensor, the measurement commenced, and the data was transmitted to the mobile phone application by Bluetooth communication (Fig. 5a).

A male with an 85 kg body weight was lying on a bed, and the sensors were attached to his forearm, elbow, and heel (Fig. 5b). The detailed results of the *P*, *T*, and *Z* measurements are shown in Fig. 5c–e. The results were obtained from three sensors (Sensor #1, #2, #3) manufactured in the same manner to verify the reliability of our sensor system. The plotted values in Fig. 5c–e are the saturated data from the mobile phone after a stabilization period of 3 min.

As shown in Fig. 5c, a higher *P* was applied to the sensor areas in the order of forearm < elbow < heel, as would be expected in a real-life scenario. Here, the *P* applied to the forearm was the compressive *P* during tape attachment. A low *P* of 4.81 ± 0.36 kPa was applied to the elbow, while an high *P* of 55.29 ± 0.75 kPa was applied to the heel. The skin *T* of the healthy subjects ranged from 25 to 35 °C, as shown in Fig. 5d. The skin *T* at all areas were within the skin *T* range expected for healthy subjects. In general, because the skin *T* is higher near the heart, a higher skin *T* at the forearm and elbow than at the heel is reasonable^6^. Furthermore, fewer microvessels distributed in the heel results in a further decrease in skin *T*; for example, a person who suffers from cold hands and feet has a lower skin *T* at the heel.

Regarding *Z*, a low input frequency results in a high *Z* value due to the high resistance component of the epidermis layer, providing information about the skin's surface, while a high input frequency results in a low *Z* value due to the low resistance of the dermis layer. As shown in Fig. S1, the skin *Z* of the healthy subjects ranged from 1 M to 100 kohm at frequency of 100 Hz and 100 kohm to 10 kohm at a frequency of 10 kHz. With our *Z* sensor, the skin *Z* could be accurately measured through the formation of a conformal contact state between the skin surface and a pair of AgNW electrodes. Due to the absence of an adhesive layer in our *Z* sensor, conformal contact can be achieved when the applied *P* level is barely greater than 5 kPa, according to our previous study. Therefore, it was determined that the *Z* values obtained from the elbow and heel, to which *P* levels approximately equal to and over 5 kPa were applied, were within the range expected for healthy subjects.

To evaluate the capability of our sensor to determine the relationship between the patient body weight and their physical biosignals, subjects of various weights were recruited. *P*, *T*, and *Z* were measured at the heels of five subjects with body weights of 50, 60, 70, 80, and 85 kg (Fig. 5f–h). As shown in Fig. 5f, the *P* at the heel increased with increasing body weight. Additionally, there was a linear relationship between the *T* at the heel of each person measured by our sensors and the *T* measured by the thermocouple, indicating our *T* sensors’ accurate monitoring capability. On the other hand, the skin *Z* values of the five subjects ranged from 15 M to 200 kohm at 0.1 kHz and 200 k to 10 kohm at 10 kHz. These *Z* values were slightly higher than those expected for healthy subjects due to the thick accumulation of dead skin at the heel region, which particularly occurs in the winter. Nevertheless, the average *Z* values of the five subjects were within the normal ranges measured via EIS, as shown in Fig. S1.

In fact, *Z* values vary widely depending on the employed electrode design or measurement method, in which there are many variables, such as the type (wet/dry), material (Ag/AgCl, Au, nanomaterial), size, geometry, and arrangement of electrodes^28,29^. To date, many clinical studies have commonly used the EIS method, which is widely used for investigating transdermal drug delivery or monitoring the late recovery of osseointegration, to determine skin *Z* changes^28,29^. Therefore, the fact that the *Z* ranges measured by our wireless sensor were comparable to those measured by a sensor using the EIS method (Fig. S1) verified the efficacy of our *Z* sensor in monitoring skin conditions. In future work, we will investigate the capability of our *Z* sensor to detect Z changes by intentionally varying the skin hydration condition and will conduct analytical/numerical simulations to optimize the geometry and arrangement of the electrode design.

## Conclusion

For the early detection of decubitus ulcers, we implemented conventional analog circuitry to achieve wirelessly and continuously communicating fabric-based multifunctional bedsore sensors. The sensor values were acquired sequentially and continuously using a BLE SoC and Z-measuring IC. Every 10 s, data were transmitted to the customized mobile phone app. Because a small-sized and lightweight PCB consumes little energy for intermittent sensing, only a coin-cell battery was needed to operate the system for over 24 h.

When comparing studies on sensing skin wounds to detect decubitus ulcers (Table S1), all the reported flexible smart sensors exploiting nanotechnologies were noninvasive and disposable. However, unlike these reported sensors, our fabric-based multifunctional decubitus ulcer sensor simultaneously monitored the required skin conditions based on P, T, and Z data. Furthermore, wireless and continuous sensing was achieved with a BLE SoC, while only intermittent sensing was achieved with the near-field communication (NFC) technologies used in other works. Last, woven cotton fabric allowed our sensor to be soft for skin application, similar to hydrogel or commercial bandages, which was hardly achieved with some stiff polymers, such as PET or polyimide (PI).

We conducted a pilot test on healthy subjects to evaluate the efficacy of the wirelessly operating multifunctional sensing system when applied to the body. Our sensor successfully measured *P*, *T*, and *Z* under applied pressure conditions, and the values were in accordance with those measured by a commercially available sensor. Overcoming the practical problems pertaining to wireless, continuous, soft, noninvasive, and disposable issues, our fabric-based multifunctional decubitus ulcer sensor is expected to promptly detect the abnormal status of skin and tissue under applied pressure conditions in actual applications.

## Methods

### Fabrication of electrodes and *Z* sensors on fabric substrates

After fixing a SUS stencil mask with a 3 mm width, 3 mm spacing, and 10 ~ 15 cm length line patterns on a fabric substrate (100% cotton, 180 thread count, 60 Su in Korean units, 50 μm thick, Clubsilkbill) with a spray adhesive (3 M), silver nanowires (AgNWs, 0.63 wt% dispersed in ethanol; Novarials) were sprayed onto the fabric on a 100 °C hot plate using a spray coater. The *P* for the spray was 10 ~ 15 psi, and the distance from the nozzle to the sample was 5 ~ 10 cm. Moreover, 2 mL AgNW solution was sprayed at a 0.75 mL/min rate.

### Fabrication of the *P* sensor

AgNWs were diluted by adding ethanol at a 1:7 volume ratio. Ten microliters of diluted AgNW solution was dropped on a cotton fabric slab with dimensions of 3 mm × 7 mm using a micropipette. The film was annealed in an oven at 100 °C for 10 min. The *P*-sensing element was mounted on a AgNW electrode and fixed using Tegaderm™ film (3 M).

### Fabrication of the *T* sensor

To prepare the *T* sensor, 1.2 µL of composition ink, which was composed of MWCNTs (1 wt% in deionized water; Sigma‒Aldrich) and a PEDOT:PSS (PH1000; Heraeus) solution with a 1:9 wt% ratio, was drop-cast onto fabric placed on a 120 °C hot plate and annealed at 120 °C for 10 min. This drop-casting process was repeated to double-coat the same location with PEDOT:PSS/MWCNT solution. After coating and annealing, the area where the *T* sensor and electrode were located was covered with Tegaderm™ film.

### Fabrication of the FFC

The FFC was fabricated using an inkjet printing machine (EPSON-1170), of which the nozzle tip dropped the commercially available Ag ink on the surface-treated PET substrate (Novele™ pack).

### Characterization

The electrical resistance of the AgNW electrodes, *P*-sensing element, and *T*-sensing element was measured using a digital multimeter (DMM 6500; Keithley). EIS (SP-300; BioLogic) was used to measure the impedance of the skin with a 1 V sweeping AC voltage at frequencies ranging from 1 Hz to 1 MHz.

### Pilot test

The pilot test was conducted briefly (~ 5 min) with five healthy subjects lying on a bed with three sensors on every area, including the forearm, elbow, and heel, to verify intersample variation. We gave the participants all the necessary information about the experiment and obtained the participant's consent before the test. The measurements performed in this study were based on the confirmation from the Yonsei University IRB center that the IRB approval was not necessary for the short time, simple attachment of the sensor on the healthy subjects, and the tests did not induce any skin irritation. The sensing data were collected through a customized mobile phone app. The weight, thermocouple (K type), and Ag/AgCl electrodes (2223H; 3 M) were employed as a commercially available standard. while sleeping on amattresswith a pair of reader antennas.

## Supplementary Information

Supplementary Information.

## Data Availability

The data that support the findings of this study are available from the corresponding author upon reasonable request.
