# Supplementary material for: A fabric-based wearable sensor for continuous monitoring of decubitus ulcer of subjects lying on a bed
Source: Sci Rep. 2023 Apr 8;13:5773. doi: 10.1038/s41598-023-33081-7 (PMC10082782; doi:10.1038/s41598-023-33081-7)
Supplement: Supplementary file 1 — Supplementary Information. [file 41598_2023_33081_MOESM1_ESM.docx]

**Supporting information**

*Soyeon Lee*^‡^*, Seung-Rok Kim*^‡^*, Kun-Hoo Jeon, Jun-Woo Jeon, Ey-In Lee, Jiwan Jeon, Je-Heon Oh, Ju-Hyun Yoo, Hye-Jun Kil, and Jin-Woo Park*^*^

Department of Materials Science and Engineering, Yonsei University, 50 Yonsei-ro, Seodaemun-gu, Seoul, 03722, Korea

*Corresponding author’s contact: Email: jwpark09@yonsei.ac.kr

‡These authors contributed equally.


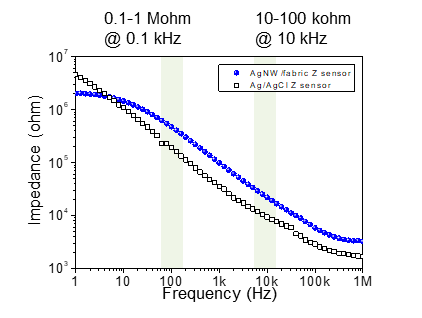


Figure S1. Skin impedance spectrum measured with the EIS method.

Table S1. Comparison of studies on skin wound sensing for preventing decubitus ulcers.

| **Multifunctional** | **Wireless** | **Continuous** | **Soft** | **Noninvasive** | **Disposable** | **Multifunctional** | **Reference** |
| --- | --- | --- | --- | --- | --- | --- | --- |
| X (*Z*) | X | X | O (Hydrogel) | O | O | X (*Z*) | [1] |
| O (*P*, *T*) | O | X (NFC) | X (PET) | O | O | O (*P*, *T*) | [2] |
| O (*P*, *T*) | O | X (NFC) | X (PI) | O | O | O (*P*, *T*) | [3] |
| O (*T*, pH, biomarkers) | O | O | X (PI) | O | O | O (*T*, pH, biomarkers) | [4] |
| O (*Z*, pH) | O | O | O (Commercial bandage) | O | O | O (*Z*, pH) | [5] |
| O (*P*, pH, bleeding) | O | O | O (Commercial bandage) | O | O | O (*P*, pH, bleeding) | [6] |
| O (*P*, *T*, *Z*) | O | O | O (Cotton fabric) | O | O | O (*P*, *T*, *Z*) | This work |

**References**

1. Swisher, S.L. et al. Impedance sensing device enables early detection of pressure ulcers in vivo. *Nat. Commun.* **6**, 6575 (2015).

2. Han, S. et al. Battery-free, wireless sensors for full-body pressure and temperature mapping. *Sci. Transl. Med.* **10**, eaan4950 (2018).

3. Oh, Y.S. et al. Battery-free, wireless soft sensors for continuous multi-site measurements of pressure and temperature from patients at risk for pressure injuries. *Nat. Commun.* **12**, 5008 (2021).

4. Gao, Y. et al. A flexible multiplexed immunosensor for point-of-care in situ wound monitoring. **7**, eabg9614 (2021).

5. Pal, A. et al. Early detection and monitoring of chronic wounds using low-cost, omniphobic paper-based smart bandages. *Biosens. Bioelectron.* **117**, 696-705 (2018).

6. Farooqui, M.F. & Shamim, A. Low Cost Inkjet Printed Smart Bandage for Wireless Monitoring of Chronic Wounds. *Sci. Rep.* **6**, 28949 (2016).
